# Molecular Expression of the Scribble Complex Genes, *Dlg*, *Scrib* and *Lgl*, in Silkworm, *Bombyx mori*

**DOI:** 10.3390/genes4020264

**Published:** 2013-05-30

**Authors:** Hai-Sheng Qi, Shu-Min Liu, Sheng Li, Zhao-Jun Wei

**Affiliations:** 1 School of Biotechnology and Food Engineering, Hefei University of Technology, Hefei 230009, China; E-Mail: hsqi0518@163.com; 2 Key Laboratory of Insect Developmental and Evolutionary Biology, Institute of Plant Physiology and Ecology, Shanghai Institutes for Biological Sciences, Chinese Academy of Sciences, Shanghai 200032, China; E-Mail: minshuliu@163.com

**Keywords:** *lethal giant larvae*, *discs large*, *scribble*, developmental expression, tissue expression, *Bombyx mori*

## Abstract

The Scribble protein complex genes, consisting of *lethal giant larvae* (*Lgl*), *discs large* (*Dlg*) and *scribble* (*Scrib*) genes, are components of an evolutionarily conserved genetic pathway that links the cell polarity in cells of humans and Drosophila. The tissue expression and developmental changes of the Scribble protein complex genes were documented using qRT-RCR method. The *Lgl* and *Scrib* genes could be detected in all the experimental tissues, including fat body, midgut, testis/ovary, wingdisc, trachea, malpighian tubule, hemolymph, prothoracic gland and silk gland. The *Dlg* gene, mainly expressed only in testis/ovary, could not be detected in prothoracic gland and hemolymph. In fat body, there were two higher expression stages of the three genes. The highest peak of the expression of the *Lgl* and *Scrib* genes in wingdisc lay at the 1st day of the 5th instar, but the *Dlg* gene was at 3rd day of 5th instar. The above results indicate that Scribble complex genes are involved in the process of molting and development of the wingdisc in the silkworm. This will be useful in the future for the elucidation of the detailed biological function of the three genes *Scrib*, *Dlg* and *Lgl* in *B. mori*.

## 1. Introduction

During development of an organism, cells exhibit different shapes from columnar epithelial cells to stellate fibroblast cells, and cell shape plays an important role for maintenance of normal cellular functions and tissue homoeostasis [1]. Cell polarity is important for cell shape maintenance, which is generally described as the asymmetric distribution of cellular constituents, and this includes membrane proteins, carbohydrates and lipids [2]. Loss of cell polarity in epithelium is commonly linked to cancer development and progression [3].

The Scribble protein complex were identified first in Drosophila as cell polarity genes consisting of *lethal giant larvae* (*Lgl*), *discs large* (*Dlg*) and *scribble* (*Scrib*), which are evolutionarily conserved components of a common genetic pathway that link the disparate functions of cell polarity and cell proliferation in epithelial cells [4]. Mutants of all three genes can induce the development of multilayered and invasive tumors in Drosophila imaginal epithelia, which are called “neoplastic” tumor-suppressor genes [5]. Intriguingly, the neoplastic tumor-suppressor mutants do not overproliferate when surrounded by wild-type tissue; instead, these mutant clones are eliminated from epithelium by cell competition [6]. *Scrib* gene encodes a membrane associated scaffolding protein, which contains 16*N*-terminal leucine-rich repeats (LRRs) and four PDZ domains (a member of the LAP family protein) [7]. *Dlg* as a scaffolding protein of the MAGUK family contains three PDZ domains, an SH3 domain, and a GUK domain [8]. Compared to *Scrib* and *Dlg*, *Lgl* is not a scaffolding protein but a WD40 repeat protein [9]. *Scrib* localizes to the adherens junction (AJ) at the basolateral region. *Dlg* binds to *Scrib* directly and is situated the same site with *Scrib*. The *Lgl* can localize to the plasma membrane after phosphorylation [10]. The *Scrib*, *Dlg*,and *Lgl* proteins are highly conserved in sequence among species, and are likely to be functionally conserved as well. In addition, mammalian *Dlg* can rescue the phenotype of a Drosophila *Dlg* mutant [11]. After the complete genome of *B. mori* was sequenced [12], the gene expression and regulation was widely analyzed [13,14,15]. However, there are no reports about the genes encoding the Scribble protein complex; including *Scribble* (*Scrib*), *Discs large* (*Dlg*) and *Lethal giant larvae* (*Lgl*) are found in silkworm, *B. mori*.

In the present research, the Scribble protein complex genes (*Scrib*, *Dlg* and *Lgl*) encoding the Scribble protein complex were identified from the silkworm, *B. mori*. The tissue expression and developmental change of these three genes were studied.

## 2. Materials and Methods

### 2.1. Insects

The silkworm larvae (P50 strain) were provided by The Sericultural Research Institute, Chinese Academy of Agricultural Sciences. They were reared with mulberry leaves in the laboratory at 25 °C under 14 h light/10 h dark cycles [16]. The different tissues, including fat body (FB), midgut (MT), testis/ovary (TE/OV), wingdisc (WD), trachea (TR), malpighian tubule (MG), hemolymph (HA), prothoracic gland (PG), and silk gland (SG), were dissected in 0.75% NaCl, and stored at −80 °C until use. Based on the molting of larvae of silkworm, the larvae after the 3rd molting to 4th molting were staged as the 4th instar; the larvae after the 4th molting to wandering stage were staged as the 5th instar; the wandering stage larvae were those that had ceased feeding. To analyze the developmental expression pattern of the three genes, the tissues from 10 silkworms were dissected and mixed as one sample.

### 2.2. Gene Identification

**Figure 1:**
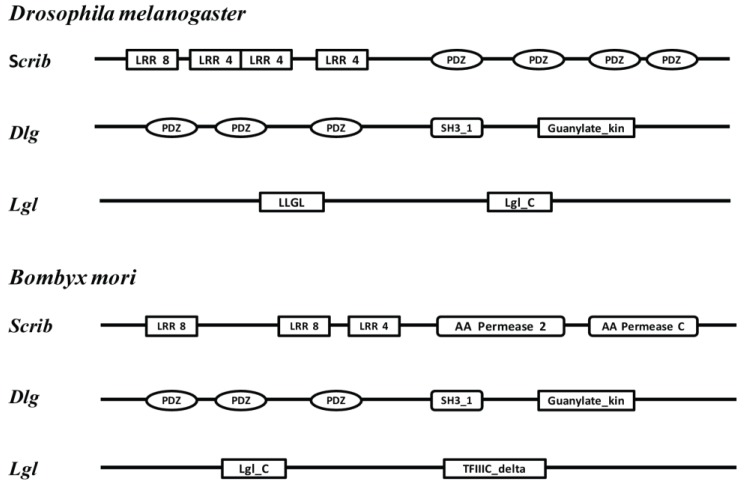
The significant domains of the scribble complex genes in *Drosophila melanogaster* and *Bombyx mori*. LRR8: Leucine rich repeat; LRR4: Leucine rich repeat (2 copies); PDZ: PDZ domain; SH3_1: SH3 domain; Guanylate kin: Guanylate kinase; LLGL: LLGL2; Lgl_C: Lethal giant larvae like, *C*-terminal; AA_permease 2: Amino acid permease; AA_permease C: *C*-terminus of AA_permease; TFIIIC delta: Transcription factor IIIC subunit delta *N*-term.

The Scribble complex genes in *B. mori* were searched in the SilkDB [17]. We used the highly conserved *Scrib* (GenBank AF190774.2), *Dlg* (GenBank M73529.1) and *Lgl* (GenBank NM_164349.3) genes of *D. melanogaster* as queries to search and identify silkworm *Scrib*, *Dlg* and *Lgl* genes by local BLASTP, with an E-value threshold of 10^−6^. The identified putative *Scrib*, *Dlg* and *Lgl* genes were validated by search of the protein database of the NCBI. The putative *Scrib*, *Dlg* and *Lgl* genes were also confirmed the sequences found in SilkDB by sequencing the related PCR products. Each gene was further analyzed by the program Pfam to identify its domains (Figure 1).

### 2.3. RNA Isolation, cDNA Synthesis

Total RNA was isolated from the different tissues of *B. mori* according to the manufacturer’s protocol of TRIzol^®^ Reagent kit (Ambion, TX, USA). Different tissue samples were homogenized with at least 1ml TRNzol Reagent in the glass homogenizer. The process of total RNA extraction and purification was carried out following the instructions including a DNase treatment. The quality of RNA was ascertained by the spectrophtometric method with an A_260_/A_280_ ratio from 1.8 to 2.0 and 1% agarose gels electrophoresis based on the integrity of 18S and 28S rRNA bands. Finally the tolal RNA was dissolved in 30 μL DEPC treated H_2_O and stored at −80 °C.

The cDNA were synthesized from 2 μg DNase treated total RNA using the M-MLV RTase cDNA Synthesis Kit (Takara, China) with the oligo(dT)_18_ primers in a 20 μL final volume according to the manufacturer’s protocol. The cDNAs were diluted 10 times and utilized to qRT-RCR experiment.

### 2.4. qRT-RCR

The qRT-RCR was performed on IQ5 Real-Time qPCR Detection System (Bio-Rad,USA) with SYBR green (Toyobo, Janpan) as dsDNA binding dyes. qRT-PCR reactions were carried out with 2 μL diluted cDNA, 10 μL SYBR^®^ Green Realtime PCR Master Mix, 0.8 μL 10 μΜ upstream primer and downstream primer and 6.4 μL ddH_2_O in a final volume of 20 μL in triplicate. The reaction was initially denatured at 95 °C for 30 s, followed by 40 cycles of denaturation at 95 °C for 5 s and annealing at 58 °C for 10 s. Melt curve analysis was performed at the end of each PCR thermal profile to assess the specificity of amplification. The primers used in this study were designed by Primer 5.0 software (Table 1).

**Table 1:** The primers used for qRT-PCR.

Gene	Primers	Sequences	GenBank
*Scrib*	*Scrib* F	5' TAGTTCAGCAACTGGAACGC 3'	BGIBMGA005373
	*Scrib* R	5' TTCTAGCCATGCGAATTGAG 3'	
*Dlg*	*Dlg* F	5' AACGCTGACGGAGAAATCTT 3'	BGIBMGA010382
	*Dlg* R	5' GAGTGTACGCGATCGTCAAT 3'	
*Lgl*	*Lgl* F	5' TCGTCTTCCGAATTACAACG 3'	BGIBMGA005570
	*Lgl* R	5' GGCACCTCTTCCTTATGCTC 3'	
*Rp49*	*Rp49 F*	5' CAGGCGGTTCAAGGGTCAATAC 3'	AY769302
	*Rp49* R	5' TGCTGGGCTCTTTCCACGA 3'	

### 2.5. Tissue-Specific Distribution and Developmental Expression of Scrib, Dlg and Lgl

The distribution of *Scrib*, *Dlg* and *Lgl* mRNAs was detected in fat body (FB), midgut (MT), testis/ovary (TE/OV), wingdisc (WD), trachea (TR), malpighian tubule (MG), hemolymph (HA), prothoracic gland (PG), and silk gland (SG) by qRT-PCR. The qRT-PCR primers are listed in Table 1. The specificity of pairs of primers was verified via the single amplification with expected size in normal PCR amplification and the only peak of the melt curve line in qRT-PCR reactions. qRT-PCR data were obtained as Ct. The mRNA expression of *Scrib*, *Dlg* and *Lgl* was normalized to the *RP49* gene for the tissue distribution. Based on the tissue distribution analysis, the developmental expression of *Scrib*, *Dlg* and *Lgl* in fat body and wingdisc was carried out.

## 3. Results

### 3.1. Tissue-Specific Distribution and Developmental Expression of the Scrib Gene

**Figure 2:**
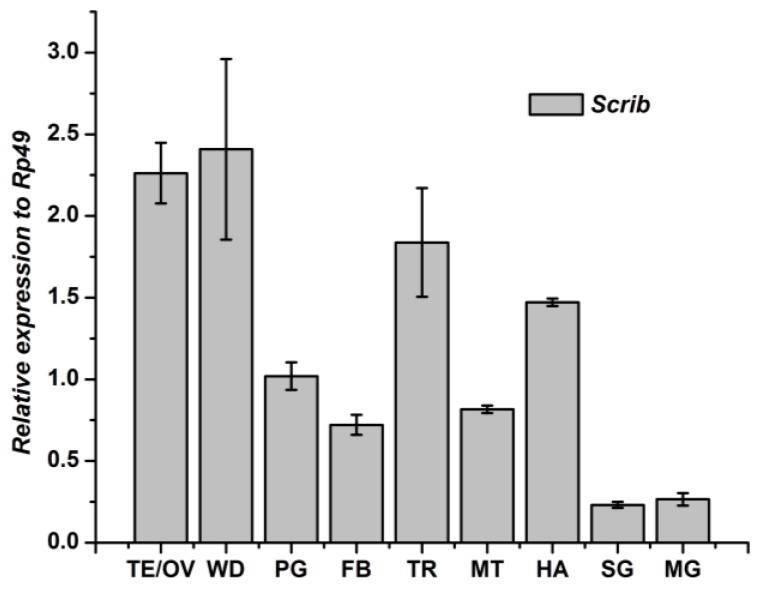
Tissue distribution of *Scrib* mRNA in *Bombyx mori*. fat body, FB; midgut, MT; testis/ovary, TE/OV; wingdisc, WD; trachea, TR; malpighian tubule, MG; hemolymph, HA; prothoracic gland, PG; silk gland, SG.

The mRNA expression patterns of the *Scrib* gene in different tissues of 2nd day of the 5th instar larval of silkworm were determined by qRT-PCR analysis (Figure 2). Our result showed that the *Scrib* gene is expressed in all the experimental tissues. The highest transcript levels of the *Scrib* gene was detected in wingdisc, and the next was in testis/ovary. Trachea and hemolymph also showed higher expression level. Malpighian tubule and silk gland showed lower level of the *Scrib* gene, with 0.27 and 0.23 fold compared to the *Rp49* gene.

**Figure 3:**
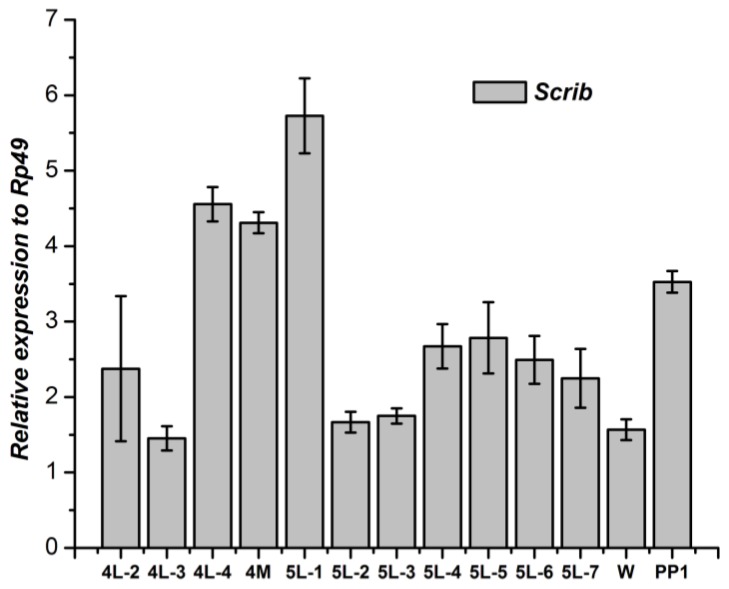
Developmental changes of *Scrib* mRNA in fat body of *Bombyx mori.* 4L-2 to 4L-4, the 2nd to 4th day of 4th instar larvae; 4M, the molting stage of 4th instar larvae; 5L-1 to 5L-7, the 1st day to 7th day of 5th instar larvae; W, wandering stage; PP1, the 1st day of pre-pupal stage.

In insects, the fat body is the central organ for energy metabolism and nutrient storage; it senses and integrates environmental signals such as nutrition and the molting hormone to regulate insect body growth. Therefore, we further detected the developmental expression profile of the *Scrib* gene in fat body of *B. mori.* From the 4th day of the 4th instar larval to the 1st day of the 5th instar, the *Scrib* gene kept higher expression, and then declined to a low level dramatically. During the middle stage of the 5th instar, the *Scrib* gene increased slightly. After the wandering phase, the *Scrib* gene increased again (Figure 3).

**Figure 4:**
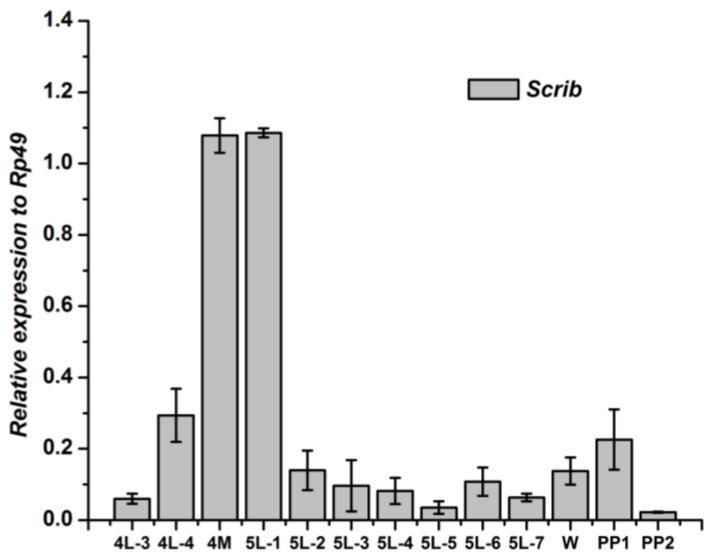
Developmental changes of *Scrib* mRNA in wingdisc of *Bombyx mori.*

Because the *Scrib* gene is expressed mainly in wingdisc (Figure 2), the developmental expression of *Scrib* mRNA was further analyzed (Figure 4). The *Scrib* gene showed the highest level from the molting stage of 4th instar to the 1st day of the 5th instar larval, but kept a low level at other stages. These results thus indicated that the *Scrib* may play a key role during the molting of 4th instar.

### 3.2. Tissue-Specific Distribution and Developmental Expression of Dlg Gene

**Figure 5:**
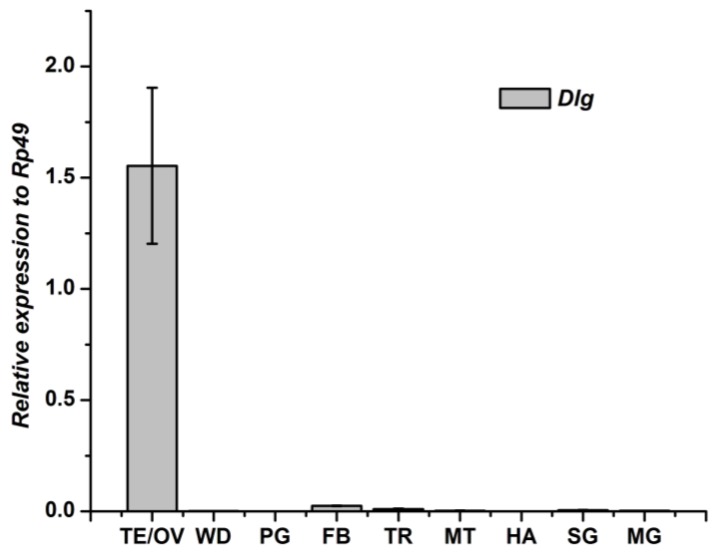
Tissue distribution of *Dlg* mRNA in *Bombyx mori.* fat body, FB; midgut, MT; testis/ovary, TE/OV; wingdisc, WD; trachea, TR; malpighian tubule, MG; hemolymph, HA; prothoracic gland, PG; silk gland, SG.

The mRNA expression patterns of *Dlg* gene in different tissues of the 2nd day 5th instar larval of silkworm were determined by qRT-PCR analysis (Figure 5). The *Dlg* gene expressed mainly in the testis/ovary, being 1.55 fold that of the *Rp49* gene. The *Dlg* gene can also be detected in fat body, trachea, silk gland, midgut and malpighian tubule. The *Dlg* mRNA was not detected in the prothoracic gland or hemolymph (Figure 5).

**Figure 6:**
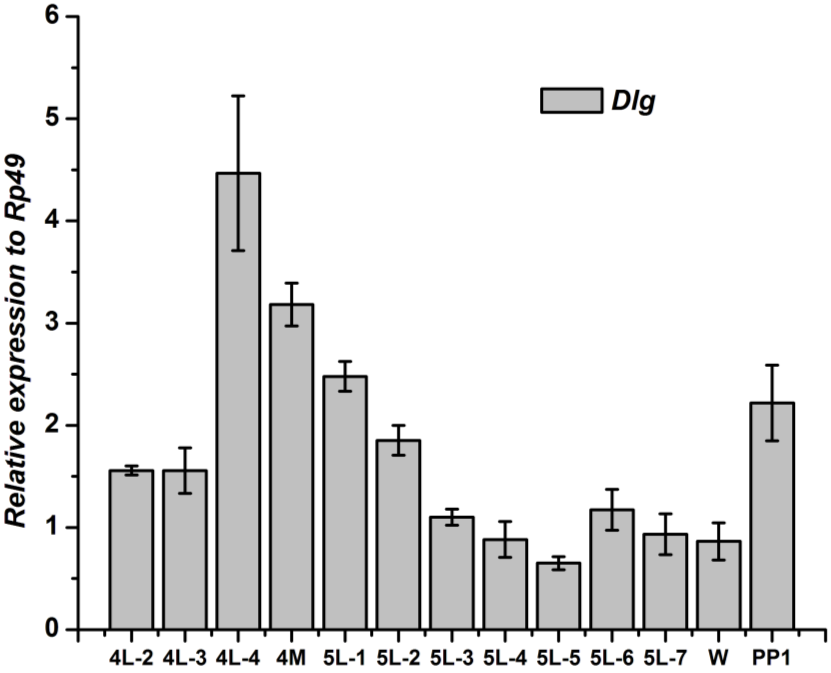
Developmental changes of *Dlg* mRNA in fat body of *Bombyx mori.*

The *Dlg* mRNA in fat body reached the peak level at the 4th day of the 4th instar larval; and then, declined continually to the wandering stage. After pupation, the *Dlg* expression in fat body increased again (Figure 6).

**Figure 7:**
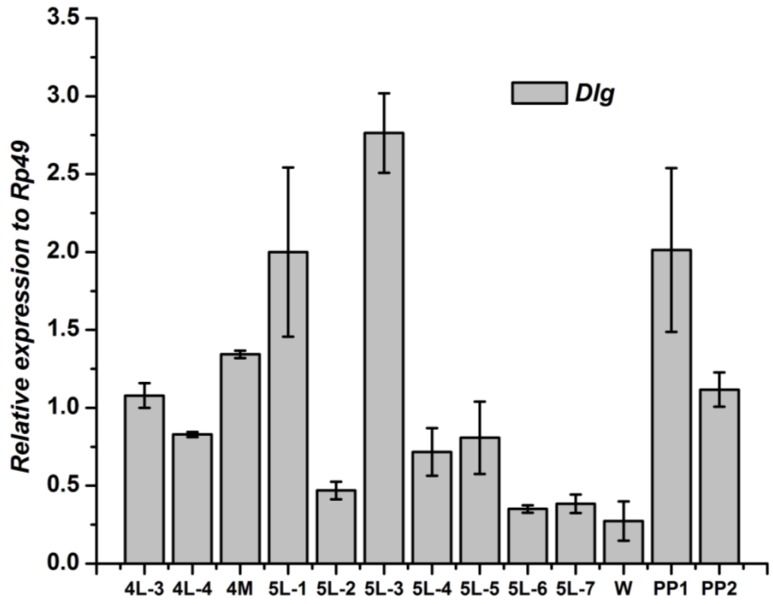
Developmental changes of *Dlg* mRNA in wingdisc of *Bombyx mori.*

The *Dlg* gene in wingdisc kept an increasing pattern from the end of the 4th instar to the 1st day of the 5th instar, and showed the highest level at the 3rd day of the 5th instar. Then, the *Dlg* gene expression declined significantly and kept a low level before pupation. After pupation, the *Dlg* expression in fat body increased again (Figure 7).

### 3.3. Tissue-Specific Distribution and Developmental Expression of Lgl Gene

**Figure 8:**
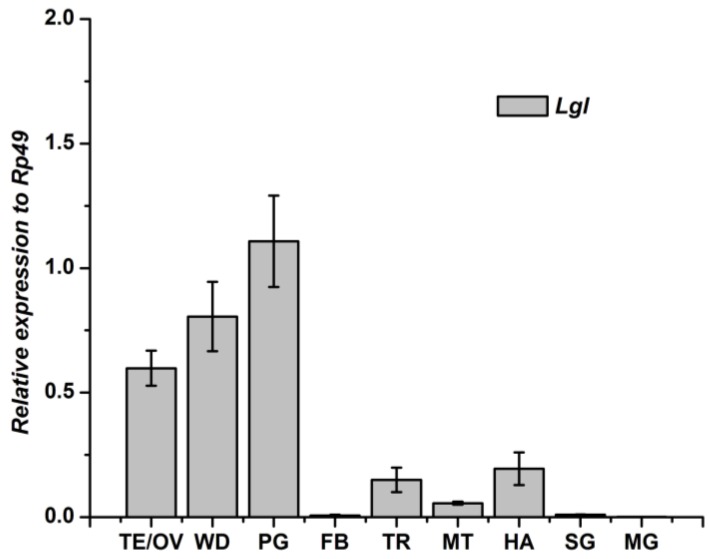
Tissue distribution of *Lgl* mRNA in *Bombyx mori.* fat body, FB; midgut, MT; testis/ovary, TE/OV; wingdisc,WD; trachea, TR; malpighian tubule, MG; hemolymph, HA; prothoracic gland, PG; silk gland, SG.

The *Lgl* gene expressed in all the experimental tissues. The highest transcript levels of *Lgl* gene was detected in the prothoracic gland, and the next highest was in the wingdisc. Testis/ovary also showed higher expression levels. The other six tissues showed very low levels of *Lgl* gene compared to the *Rp49* gene (Figure 8).

**Figure 9:**
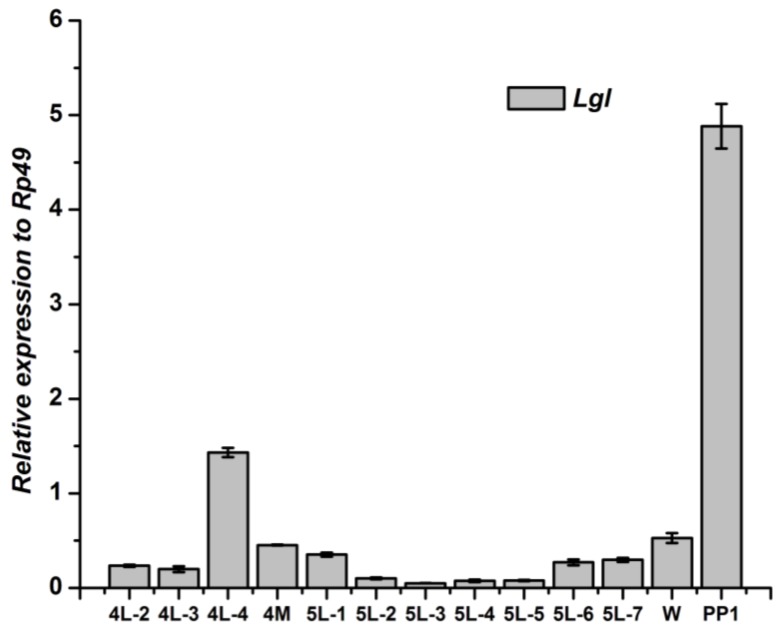
Developmental changes of *Lgl* mRNA in fat body of *Bombyx mori.*

The *Lgl* gene in fat body kept a stable low level from the 2nd to 4th instar, then, reached a relative higher peak at the 4th day of 4th instar. From the molting stage of 4th instar to the wandering stage, the *Lgl* gene in fat body maintained low expression. After pupation, the *Lgl* expression in fat body increased again (Figure 9), which is similar to the patterns of *Scrib* and *Dlg* gene in fat body.

**Figure 10:**
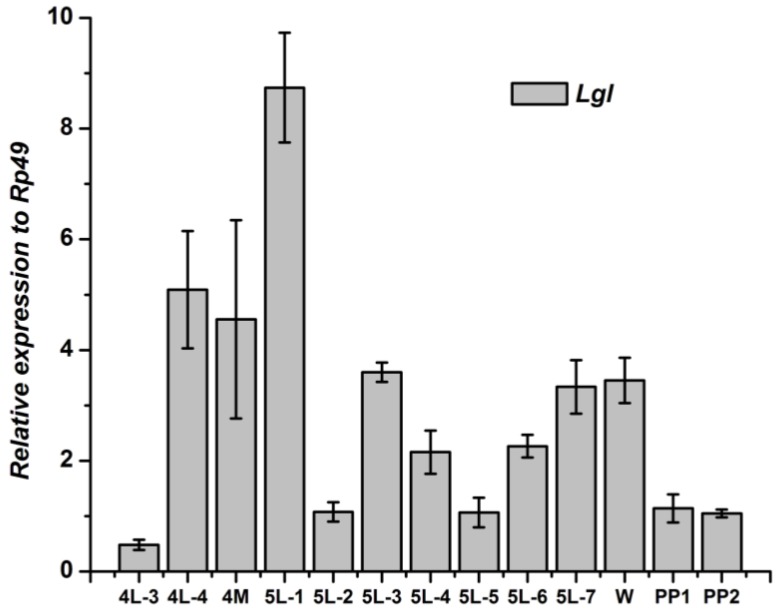
Developmental changes of *Lgl* mRNA in wingdisc of *Bombyx mori.*

The *Lgl* gene in wingdisc changed discontinuously from the 3rd day of the 4th instar to pupae. During the two molting stages, from 4th instar to the 5th and from the 5th instar to pupation, the *Lgl* gene showed a relatively higher level. The *Lgl* gene expressed highest at the 1st day of the 5th instar larva (Figure 10). After pupation, the *Lgl* in wingdisc kept a low level, which was different to that in fat body.

## 4. Discussion

In the present study, the tissue distribution and development expression of the Scribble complex genes (*Scrib*, *Dlg* and *Lgl*) in *B. mori* were performed. To date, there have been no reports about the tissue distribution of *Scrib* and *Dlg* genes in insects. Only the tissue distribution of the *Lgl* genes in *Drosophila melanogaster* has been reported in previous research [18]. In our results, The *Lgl* gene mainly expressed in prothoracic gland, wingdisc and testis/ovary in *B. mori*, can also be detected in fat body, midgut, trachea, malpighian tubule, hemolymph and silk gland, and these showed some difference in the distribution with *D. melanogaster.* Western blot analysis showed that the *Lgl* gene mainly expressed in brain and wingdisc, the expression in fat body was weaker, but could not be detected in midgut and hemolymph in *D. melanogaster* [18]. The difference of the *Lgl* distribution between two insects might be attributed to that the qRT-RCR used in the present research is more sensitive than Western blotting.

The development expressions of the three genes of Scribble complex (*Scrib*, *Dlg* and *Lgl*) of *B. mori* in the two tissues were different to each other. Although the expression level of the three genes in fat body were different, the two higher expression stages were similar, one was in the stage from the 4th day of the 4th instar to the 1st day of the 5th instar, the other was the 1st day after pupation. The two stages are relative to the molting of the silkworm, which indicated that the three genes of Scribble complex in fat body may play some important roles in the silkworm molting process. The developmental profiles of the three genes in wingdisc were different to that in the fat body. The highest peak of the expression of *Lgl* and *Scrib* gene in wingdisc lay at the 1st day of the 5th instar, but the *Dlg* gene was at the 3rd day of the 5th instar. There was a relatively higher expression peak to the *Dlg* gene in the wingdisc after pupation, but the *Lgl* and *Scrib* genes kept a low expression at this stage. The above differences indicated that the three genes may play different roles in the wingdisc in silkworm. The expression profiles related to the function of genes, but the function of the three genes of Scribble complex (*Scrib*, *Dlg* and *Lgl*) in *B. mori* still remains unclear, and the further research about the function is necessary in the future.

## 5. Conclusions

Within the experimental tissues, The *Scrib*, *Dlg* and *Lgl* mainly expressed in the testis/ovary, wingdisc, and prothoracic gland in *B. mori*, respectively. There were two relatively higher expression stages of the three genes in fat body of *B. mori*. The highest peak of the expression of the *Lgl* and *Scrib* genes in wingdisc lay at the 1st day of the 5th instar, but the *Dlg* gene was at the 3rd day of the 5th instar.
